# *Belonolaimus Longicaudatus* Host Status and Pathogenicity on Sweetpotato

**DOI:** 10.2478/jofnem-2022-0019

**Published:** 2022-07-02

**Authors:** Zane J. Grabau, Chang Liu, Rebeca Sandoval-Ruiz, Wendy Mussoline

**Affiliations:** 1 Entomology and Nematology Department, University of Florida, Gainesville, FL U.S.A; 2 Flagler County Extension, University of Florida, Bunnell, FL U.S.A

**Keywords:** *Belonolaimus longicaudatus*, corn, *Crotalaria juncea*, host, *Ipomoea batatas*, management, sting nematode, sunn hemp, sweetpotato, *Zea mays*

## Abstract

Sting nematode is acutely damaging to a wide range of crops and is relatively common in sandy soils in the southeastern United States. Sweetpotato is an important crop in this region, and its production may be expanding to localities where sting nematode is an important pest. Despite this, the relationship between sweetpotato and sting nematode is not well-defined. Therefore, the objectives of this study were to assess (1) the relative host status of sweetpotato for sting nematode and (2) damage potential of sting nematode on sweetpotato in repeated greenhouse experiments. A known sting nematode host (field corn), a known poor host (sunn hemp), and sweetpotato cultivars susceptible (‘Beauregard’) and resistant (‘Covington’) to southern root-knot nematode were challenged with sting nematode. In both trials, field corn supported greater final soil sting nematode abundances than sunn hemp or either sweetpotato cultivar. Based on the average reproductive factor, field corn was confirmed as a susceptible host, whereas sunn hemp and sweetpotato were poor hosts. Sting nematode did not impair the growth of any crop, suggesting greenhouse conditions were not conducive to damage since field corn sustains damage in field conditions. These results suggest that sunn hemp and sweetpotato could be useful rotation crops for managing sting nematode, but future work is needed to assess sting nematode pathogenicity on these crops under field conditions.

Sting nematode (*Belonolaimus longicaudatus* Rau, 1958) is an important plant-parasitic nematode in warm climate regions, particularly the southeastern United States (Watson and Desaeger, 2019; Grabau *et al*., 2019). It damages a wide range of crops including cabbage (*Brassica oleracea* var. *capitata* L.), corn (*Zea mays* L.), cotton (*Gossypium hirsutum* L.), potato (*Solanum tuberosum* L.), strawberry (*Fragaria* x *ananassa* Duchesne), peanut (*Arachis hypogea* L.), citrus, and turfgrass (Rhoades, 1971; Rhoades, 1977; Rhoades, 1978; Rhoades, 1985; Duncan *et al*., 1996; Crow *et al*., 2000a, 2000c; Koenning *et al*., 2006; Aryal *et al*., 2015; Kutsuwa *et al*., 2015; Watson and Desaeger, 2019; Grabau *et al*., 2019). Sting nematode is acutely damaging to most of these crops even at low initial populations (Crow *et al*., 2000a, 2000c). For example, in cotton, initial populations of 100 or more sting nematodes/130 cm^3^ soil effectively eliminated yield (Crow *et al*., 2000a). In potato, initial populations as little as two or three sting nematodes/130 cm^3^ soil were the economic threshold where expected damage warranted management, and each sting nematode detected was linked to a 199 kg/ha reduction in potato yield (Crow *et al*., 2000c).

Despite its virulence, the host status and susceptibility to damage of certain crops by sting nematode have not been reported. To our knowledge, this information is not known for sweetpotato (*Ipomoea batatas* (L.) Lam.). In the United States, sweetpotato is an important crop with 63,000 ha of sweetpotato worth 726 million US dollars grown in 2020 (NASS-USDA, 2021). Its production is concentrated in North Carolina, comprising 67% of acreage in 2020, with five other states, namely, Arkansas, California, Florida, Louisiana, and Mississippi comprising the rest of the US production (NASS-USDA, 2021). Sting nematode has been reported throughout the Southeast (Rhoades, 1978; Zeng *et al*., 2012; da Silva *et al*., 2019) and California (Mundo-Ocampo *et al*., 1994). Despite that, the paucity of information on the sweetpotato–sting nematode relationship may be due, in part, to the limited sting nematode prevalence in sweetpotato-producing areas of many of these states. In sweetpotato-producing states, sting nematode has been studied and reported primarily on turfgrass (Mundo-Ocampo *et al*., 1994; Zeng *et al*., 2012), rather than agricultural crops, and sting nematode prefers sandy soils (Mashela *et al*., 1991), which may restrict its distribution. However, there are efforts to produce sweetpotato in locales where sting nematode is a predominant nematode threat, for instance, Northeast Florida, which makes exploring this nematode–plant relationship especially pertinent.

In Northeast Florida, potato and cabbage are the most important cash crops. Crops are grown annually, with potato often rotated with a summer sorghum–sudangrass (*Sorghum x drummondii* (Nees ex Steud.) Millsp. & Chase) cover crop, and cabbage often rotated with a spring field corn crop. All these crops are susceptible to sting nematode, which is one reason that nematode is so prevalent in this region (Rhoades, 1977; Rhoades, 1985; Crow *et al*., 2000b; Crow *et al*., 2001). In recent years, sunn hemp (*Crotalaria juncea* L.), a poor host of sting nematode (Braz *et al*., 2016), has been grown as a summer cover crop on some acreage in this area, in part, to help manage sting nematode.

Sweetpotato production is being explored as an alternative to traditional cash crops in the region in an effort to diversify production, mitigate risk, and improve profit margins. Understanding the relationship between sting nematode and sweetpotato is important for sweetpotato production to expand into Northeast Florida and other locales where this nematode is prevalent. Sweetpotato host status for sting nematode influences its fit in rotation with current crops. Determining sting nematode pathogenicity (damage) to sweetpotato is also an initial step in determining nematode management practices needed when cultivating this crop in fields infested with sting nematode. Among sweetpotato cultivars, there is resistance to *Meloidogyne incognita* (Kofoid and White, 1919) Chitwood, 1949 (southern root-knot nematode), the most widespread nematode problem in sweetpotato. For example, ‘Covington’ exhibits resistance to southern root-knot nematode (Yencho *et al*., 2008) and is the predominant conventional (orange-fleshed) variety grown in Florida. Cultivars susceptible to southern root-knot nematode, such as ‘Beauregard’ (Yencho *et al*., 2008), are also grown. There is no prior knowledge to suggest there would be cross-resistance against sting nematode, but it is prudent to test one southern root-knot nematode-susceptible cultivar and one resistant cultivar in this screening against sting nematode.

Therefore, the objectives of this study were to (1) assess the host status of sweetpotato for sting nematode and (2) assess the pathogenicity of sting nematode to sweetpotato based on greenhouse experiments.

## Materials and Methods

### Experimental design

These objectives were investigated in repeated greenhouse experiments conducted in Gainesville, FL. The experiment was arranged in a randomized complete block design with five replicates and two fully crossed factors: crop and sting nematode inoculation rate. Four host crops were used: (1) field corn, (2) sunn hemp, sweetpotato cultivars (3) ‘Beauregard’, and (4) ‘Covington’. For field corn, the cultivar NK 1573_3330 (Syngenta Crop Protection AG, Basel, Switzerland) was used. For sunn hemp, the cultivar Crescent Sun (Tropical Seeds, LLC, Miami, FL) was used. Field corn was selected as a known sting nematode host (Rhoades, 1978; Todd, 1989), and sunn hemp as a reported poor host (Braz *et al*., 2016). As previously described, ‘Covington’ and ‘Beauregard’ were selected for their varying susceptibilities to southern root-knot nematode. Sting nematode inoculation rates were uninoculated, medium, and high. In the first trial (Trial 1), the medium and high inoculation rates were 26 and 157 sting nematodes (mixture of males, females, and juveniles) per pot, respectively. In the second trial (Trial 2), the medium and high inoculation rates were 40 and 240 sting nematodes per pot, respectively. The primary purpose of varying the inoculation rates was to assess sting nematode damage to crops. The secondary purpose was to help ensure a representative assessment of the host status of the crops because if the crop growth is severely impaired from high sting nematode inoculation rates, it could result in misleading low final sting nematode populations.

### Trial establishment

Trials were established in a greenhouse at the University of Florida in Gainesville, FL, in summer 2021. Crops were planted on 28 May (Trial 1) and 1 June (Trial 2) into 15-cm-diameter clay pots filled with 1,000 cm^3^ autoclaved field soil. The field soil was a Chipley–Foxworth–Albany complex (91% sand, 6.8% silt, and 2.4% clay with 1.7% organic matter) obtained from the University of Florida North Florida Research and Education Center near Live Oak, FL. Field corn and sunn hemp were planted with three seeds and thinned to one seed per pot 7 days after planting (DAP). Sweetpotato was planted with one slip per pot. Slips are cuttings of plant stems grown from seed tubers and are standard commercial planting materials. Sweetpotato slips were obtained from the North Carolina State University breeding program fields, transported in cardboard boxes with adequate moisture, and stored under moist conditions until transplanting. The trial was established, and the slips were transplanted 1 day after arrival in Trial 1 and 4 days after arrival in Trial 2.

### Sting nematode inoculation and plant maintenance

Sting nematodes were inoculated into pots at the previously indicated rates at 12 and 16 DAP in Trials 1 and 2, respectively. For inoculation, three holes of 2 cm depth were formed around each plant, and sting nematode inoculum was pipetted equally into each hole in a total solution of 12 mL per pot. Sting nematode inoculum was obtained from a culture maintained on St. Augustine grass ‘FX-313’ (*Stenotaphrum secundatum* (Walt.) Kuntze) and originally collected from ‘Tifway’ bermudagrass (*Cynodon dactylon* (L.) Pers.) in Sun City, FL. Sting nematodes for inoculation were extracted from the culture using the decanting and sieving method using a 400-mesh standard sieve (Cobb, 1918), followed by a modified Baermann method (McSorley and Frederick, 1991), with nematodes collected using a 500-mesh standard sieve, which was selected to maximize sting nematode viability. Nematode inoculum was quantified using a Zeiss (New York, USA) Primovert light microscope, stored at 5°C until inoculation, and was inoculated within 2 days of extraction. The plants were maintained for 70 *d*ays and 68 days after inoculation (DAI) in Trials 1 and 2, respectively, before the trials were terminated. The plants were watered daily by hand and maintained without supplemental light.

### Data collection

In each trial at 30 DAI, the plant height and number of leaves per plant were assessed. The plant height, leaves per plant, fresh shoot weight, and fresh root weight were assessed at trial termination. Soil nematode populations in each pot were quantified at the termination of each trial. To quantify soil sting nematode abundances, the soil was mixed manually, and nematodes were extracted from 100 cm^3^ soil using the sucrose centrifugation method (Jenkins, 1964). Sting nematodes were quantified morphologically by microscopy. In addition to soil abundances, at medium and high inoculation rates, reproductive factor was calculated. First, total sting nematode abundance per pot was calculated by multiplying the abundance in 100 cm^3^ soil by 10 since there was 1,000 cm^3^ soil in each pot. Then reproductive factor was calculated by dividing final soil nematode population per pot by the initial inoculum per pot. Typically, a crop that supports a reproductive factor >1 is considered a host, whereas a crop that supports a reproductive factor <1 is considered a poor or non-host (Timper and Hanna, 2005).

### Statistical analysis

All variables were analyzed separately for each trial because there were significant (ANOVA, *P <* 0.05) treatment-by-trial interactions in a preliminary analysis of sting nematode abundances. For each trial, sting nematode abundance and reproductive factor were analyzed by two-way ANOVA, with crop and inoculation rate as factors. If there were significant crop–inoculation rate interactions, crop effects on sting nematode parameters were analyzed separately with each inoculation rate. If crop effects were significant (*P <* 0.05), crop treatments were separated by Fisher’s protected LSD (a = 0.05). For crop growth parameters (number leaves, plant height, shoot weight, and root weight), the inoculation rate effect was analyzed for each crop using one-way ANOVA since direct growth comparisons among crops were not of scientific interest for this study. If inoculation rate effects on crop growth parameters were significant (*P <* 0.05), inoculation rate treatments were separated by Fisher’s protected LSD (a = 0.05). Before completing ANOVA, response variables were transformed, if needed, to meet assumptions of homogeneity of variance using Levene’s test (Levene, 1960) and normality of residuals based on graphing (Cook and Weisburg, 1999). In Trial 1, soil sting nematode abundances were transformed by square root to meet model assumptions for the full two-way ANOVA model only. For all other variables and models, transformation was not necessary. Analyses were conducted in R statistical software (version 3.4.4, The R Foundation for Statistical Computing, Vienna, Austria).

## Results

### Crop host status for sting nematode

**Figure 1:**
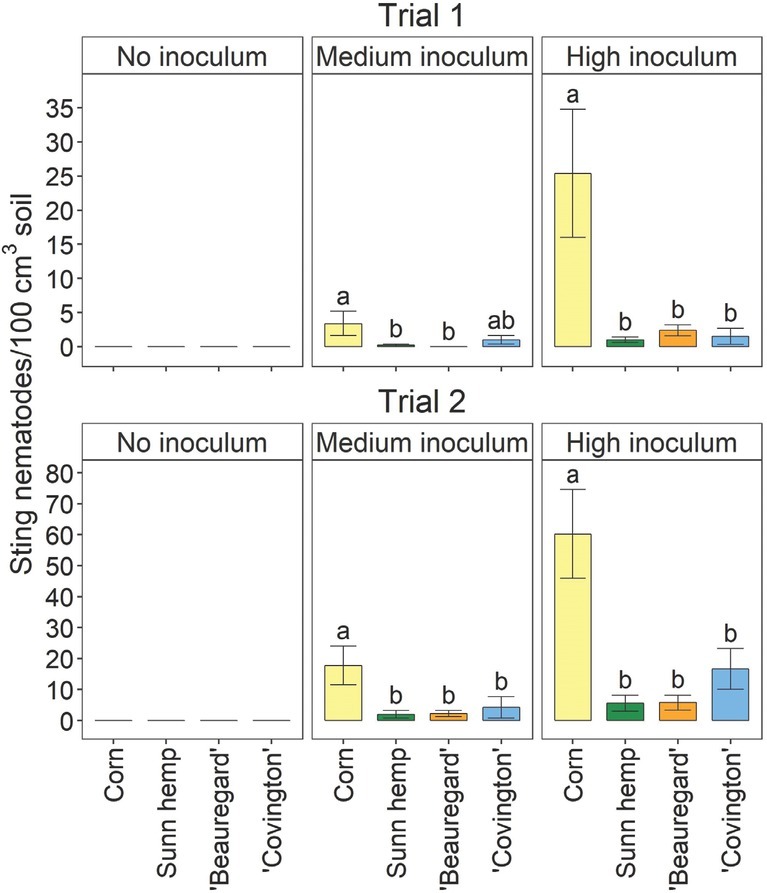
Soil sting nematode abundances at the termination of the greenhouse experiment as influenced by sting nematode (*Belonolaimus longicaudatus* Rau, 1958) inoculation rate and crop type including sweetpotato (*Ipomoea batatas* (L.) Lam.) cultivars, corn (*Zea mays* L.), and sunn hemp (*Crotalaria juncea* L.). In subfigure titles, “no inoculum,” “medium inoculum,” and “high inoculum” indicate non-inoculated, medium (26 and 40 sting nematodes per pot in Trials 1 and 2, respectively), and high rates of sting nematode inoculation (157 and 240 sting nematodes per pot in Trials 1 and 2, respectively). Letters indicate significant (Fisher’s protected LSD, *P <* 0.05) differences among crops within inoculation rate and trial. Values are means (N = 5) and standard errors.

**Figure 2:**
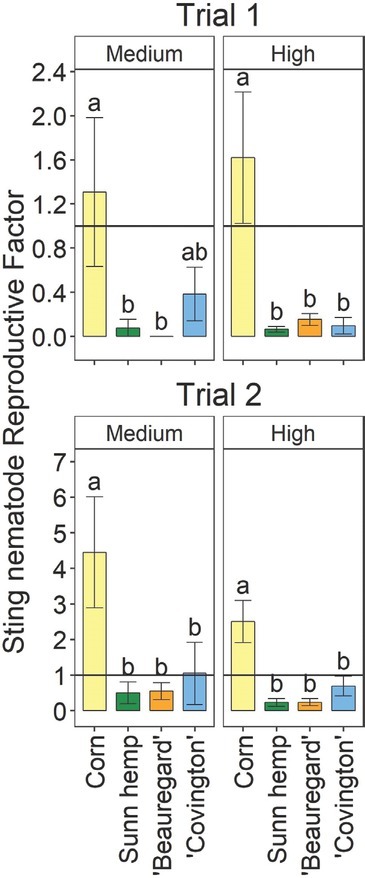
Sting nematode (*Belonolaimus longicaudatus* Rau, 1958) reproductive factor (final divided by initial soil abundances) at the termination of the greenhouse experiment as influenced by crop type – including sweetpotato (*Ipomoea batatas* (L.) Lam.) cultivars, corn (*Zea mays* L.), and sunn hemp (*Crotalaria juncea* L.) – within sting nematode inoculation rate. The horizontal line in each pane indicates the reproductive factor is equal to 1, which generally delineates the host status. In subfigure titles, “medium” and “high” indicate medium (26 and 40 sting nematodes per pot in Trials 1 and 2, respectively) and high rates of sting nematode inoculation (157 and 240 sting nematodes per pot in Trials 1 and 2, respectively). Letters indicate significant (Fisher’s protected LSD, *P* < 0.05) differences among crops within inoculation rate and trial. Values are means (N = 5) and standard errors.

For both trials, there were significant crop– inoculation rate effects on sting nematode parameters (data not shown), so the parameters were analyzed separately by inoculation rate. For both trials, soil sting nematode abundances were significantly affected by crop at the medium and high inoculation rates, but no nematodes were detected in the uninoculated treatment (Fig. 1). At both medium and high inoculation rates, soil sting nematode abundances were generally significantly greater for corn than for any other crop in both trials (Fig. 1). The only exception was that in Trial 1, at the medium inoculation rate, soil sting nematode abundances for ‘Covington’ were not significantly different from those for corn or the other crops. Sting nematode reproductive factor followed the same trends as soil sting nematode abundances (Fig. 2). In both trials and inoculation rates, sting nematode reproductive factor was >1 for corn, indicating a susceptible host, although values were numerically greater in Trial 2 than in Trial 1 (Fig. 2). Sunn hemp and ‘Beauregard’ had reproductive factors <1 in each trial, as well as each inoculation rate. ‘Covington’ had a reproductive factor <1, except in Trial 2 at the medium inoculation rate, when reproductive factor was at 1 (Fig. 2). Across both trials and inoculation rates, sting nematode reproductive factors were 2.5, 0.6, 0.2, and 0.2 for field corn, ‘Covington’, ‘Beauregard’, and sunn hemp, respectively.

### Sting nematode impacts on crop growth

Sting nematode had relatively little impact on crop growth. Sting nematode inoculation rate did not significantly affect ‘Beauregard’ or ‘Covington’ plant height at any time (Table 1). At 30 DAI in Trial 2, the corn plant height was greater at the medium rate than at the uninoculated or high inoculation rate but was not affected at any other time (Table 1). In Trial 1, the sunn hemp plant height was significantly greater at the medium rate than at the uninoculated or high rate at both 30 DAI and termination but was not significantly affected in Trial 2. Sting nematode inoculation rate did not have a consistent effect on leaves per plant at 30 or termination DAI in either trial (Table 2). In Trial 2 at 30 DAI, there were significantly more corn leaves per plant at the medium rate than at the uninoculated or high inoculation rate. In Trial 1 at 30 DAI, there were significantly more ‘Beauregard’ leaves per plant at the high than at the uninoculated rate. There were no other significant effects on corn or ‘Beauregard’ leaf counts and no effects on ‘Covington’ or sunn hemp leaf counts.

**Table 1:** Plant height (cm) of sweetpotato (*Ipomoea batatas* (L.) Lam.) cultivars, corn (*Zea mays* L.), and sunn hemp (*Crotalaria juncea* L.) under various sting nematode (*Belonolaimus longicaudatus* Rau, 1958) inoculation rates in greenhouse experiments.

	Corn^a^	Sunn hemp	‘Beauregard’	‘Covington’
Inoculation rate^c^		Plant height 30 DAI: Trial 1^b^		
Non-inoculated	42.6	34.5 b	41.0	26.2
Medium	32.4	50.6 a	26.0	39.0
High	38.4	39.0 b	35.2	36.5
Inoculation rate		Plant height 30 DAI: Trial 2		
Non-inoculated	44.9 b	36.3	27.5	19.0
Medium	37.2 a	32.9	27.8	23.9
High	48.1 b	27.1	26.4	25.2
Inoculation rate		Plant height 70 DAI: Trial 1		
Non-inoculated	39.8	57.0 b	67.1	38.5
Medium	40.5	85.5 a	47.8	39.4
High	40.4	56.9 b	76.1	43.0
Inoculation rate		Plant height 68 DAI: Trial 2		
Non-inoculated	42.5	60.5	44.6	21.2
Medium	45.4	55.7	52.9	31.3
High	46.7	49.6	60.4	31.2

a Values are means of five replicates. Letters within a crop, parameter, and run indicate significant differences (Fisher’s protected LSD, *P* < 0.05). Values with no letters are not significantly different (ANOVA, *P* < 0.05). ^b^DAI is days after inoculation. ^c^Medium and high inoculation rates were 26 and 157 sting nematodes per pot, respectively, in Trial 1, and 40 and 240 sting nematodes per pot, respectively, in Trial 2. DAI, days after inoculation.

**Table 2:** Leaves per plant for sweetpotato (*Ipomoea batatas* (L.) Lam.) cultivars, corn (*Zea mays* L.), and sunn hemp (*Crotalaria juncea* L.) at various sting nematode (*Belonolaimus longicaudatus* Rau, 1958) inoculation rates in greenhouse experiments.

	Corn^a^	Sunn hemp	‘Beauregard’	‘Covington’
Inoculation rate^c^		Leaves per plant 30 DAI: Trial 1^b^		
Non-inoculated	5	13	7 b	5
Medium	7	13	8 ab	6
High	5	11	9 a	6
Inoculation rate		Leaves per plant 30 DAI: Trial 2		
Non-inoculated	6 b	13	9	6
Medium	7 a	12	9	8
High	6 b	12	10	7
Inoculation rate		Leaves per plant 70 DAI: Trial 1		
Non-inoculated	11	18	10	10
Medium	10	23	10	9
High	10	16	13	9
Inoculation rate		Leaves per plant 68 DAI: Trial 2		
Non-inoculated	9	21	11	6
Medium	9	19	11	9
High	10	19	14	9

a Values are means of five replicates. Letters within a crop, parameter, and run indicate significant differences (Fisher’s protected LSD, *P* < 0.05). Values with no letters are not significantly different (ANOVA, *P* < 0.05). ^b^DAI is days after inoculation. ^c^Medium and high inoculation rates were 26 and 157 sting nematodes per pot, respectively, in Trial 1, and 40 and 240 sting nematodes per pot, respectively, in Trial 2. DAI, days after inoculation.

**Figure 3:**
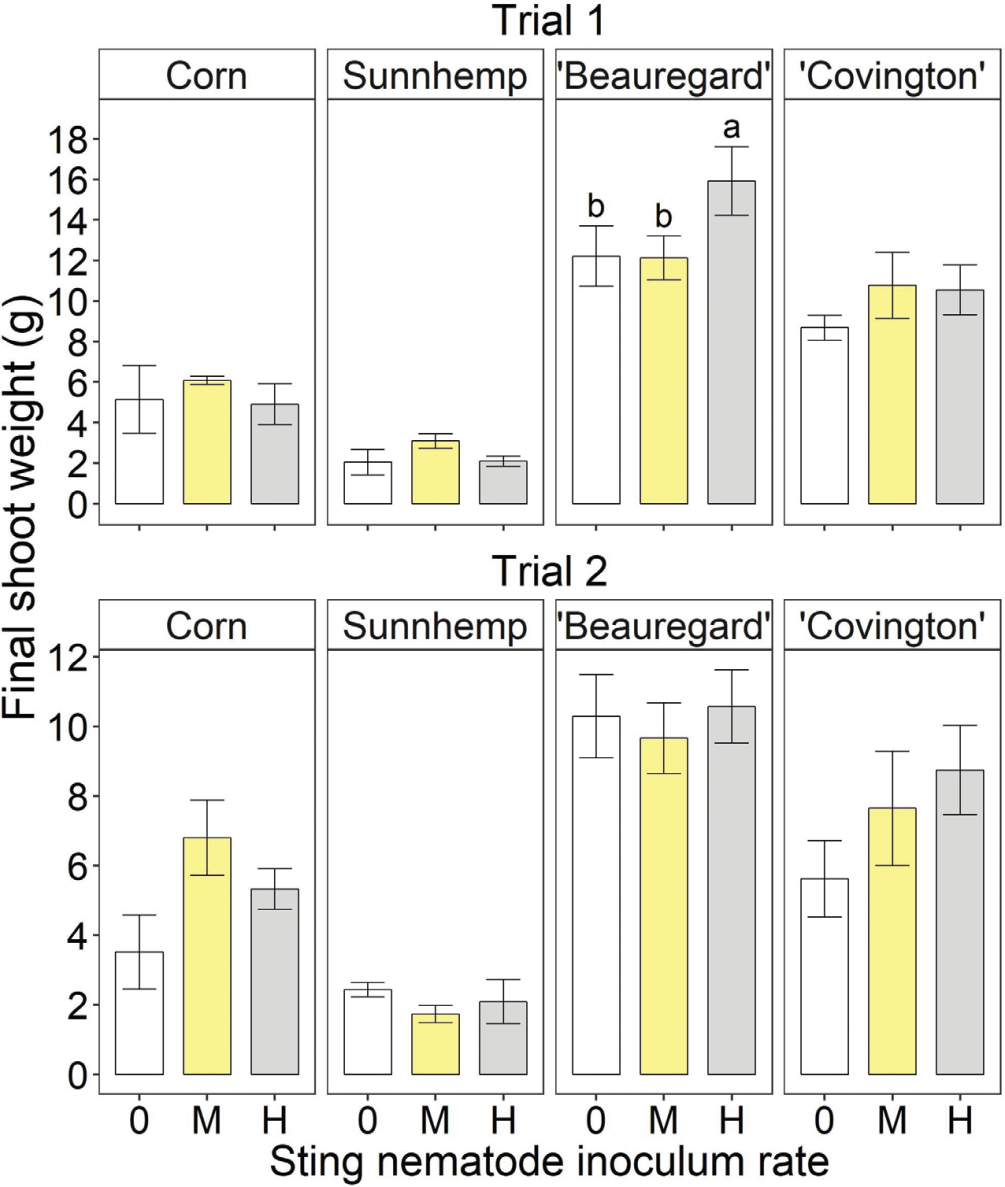
Final shoot weight at the termination of the greenhouse experiment for different crops – including sweetpotato (*Ipomoea batatas* (L.) Lam.) cultivars, corn (*Zea mays* L.), and sunn hemp (*Crotalaria juncea* L.) – as influenced by sting nematode (*Belonolaimus longicaudatus* Rau, 1958) inoculation rate. “0,” “M,” and “H” indicate non-inoculated, medium (26 and 40 sting nematodes per pot in Trials 1 and 2, respectively), and high rates of sting nematode inoculation (157 and 240 sting nematodes per pot in Trials 1 and 2, respectively). Letters indicate significant (Fisher’s protected LSD, *P <* 0.05) differences among inoculation rates within crop and trial. Values are means (N = 5) and standard errors.

**Figure 4:**
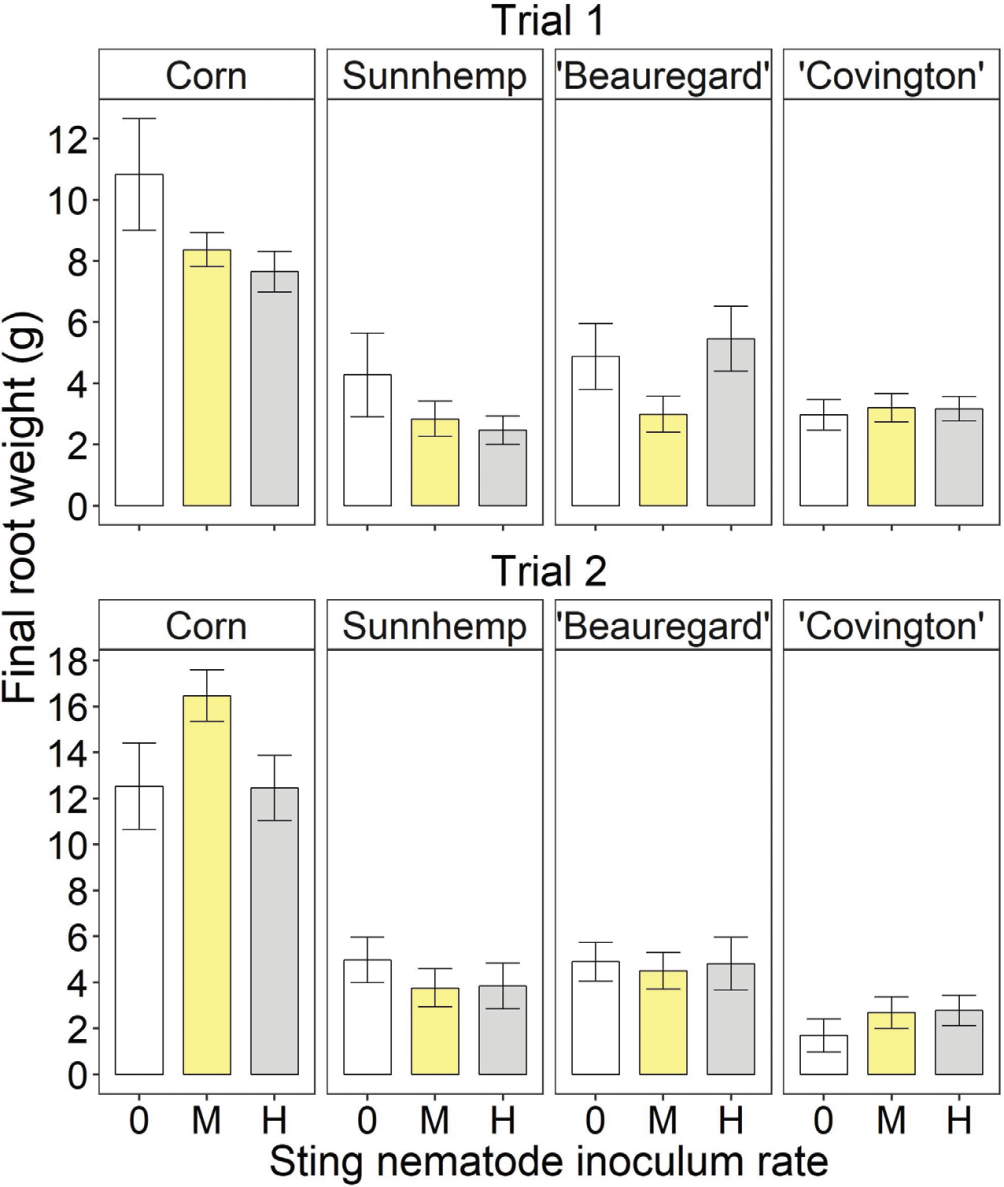
Final root weight at the termination of the greenhouse experiment for different crops – including sweetpotato (*Ipomoea batatas* (L.) Lam.) cultivars, corn (*Zea mays*, L.), and sunn hemp (*Crotalaria juncea* L.) – as influenced by sting nematode (*Belonolaimus longicaudatus* Rau, 1958) inoculation rate. “0,” “M,” and “H” indicate non-inoculated, medium (26 and 40 sting nematodes per pot in Trials 1 and 2, respectively), and high rates of sting nematode inoculation (157 and 240 sting nematodes per pot in Trials 1 and 2, respectively). Letters indicate significant (Fisher’s protected LSD, *P <* 0.05) differences among inoculation rates within crop and trial. Values are means (N = 5) and standard errors.

There were no significant inoculation rate effects on corn, sunn hemp, or ‘Covington’ final shoot weight in either trial (Fig. 3). For ‘Beauregard’, the final shoot weight was significantly greater at the high rate than at the medium or uninoculated rate in Trial 1 but was not affected in Trial 2. There were no significant inoculation rate effects on the final root weight for any crop in either trial (Fig. 4).

### Observation of sting nematode damage to crop roots

Qualitatively, minor indications consistent with sting nematode damage (Grabau and Vann, 2021), including root stunting, pruned lateral roots, and infrequent root tip necrosis, were observed on some corn plants. No obvious indications of nematode damage were observed on sunn hemp or sweetpotato roots. No sweetpotato tubers had formed by the termination of either trial, so there was no observation of potential symptoms on tubers. No formal damage ratings were made because indications were minor and relatively infrequent.

## Discussion

Sweetpotato and sunn hemp were less suitable hosts for sting nematode than field corn, a known host for sting nematode (Rhoades, 1978; Timper and Hanna, 2005). Overall, sweetpotato was a poor host of sting nematode as the average reproductive factor for each cultivar was <1, which is often considered the threshold for host status. ‘Covington’ sweetpotato may have supported sting nematode reproduction to some extent based on soil abundances and a reproductive factor near 1 at the medium inoculation rate in Trial 2. In the future, testing in field conditions for a longer duration could help clarify this. Reproductive factor values may be inflated moderately in the greenhouse compared with field conditions as abundant roots and controlled environment conditions in the greenhouse often favor nematode reproduction (Timper *et al*., 2006). Additionally, sting nematode inoculation rates were less (26 to 240 nematodes per pot) in this greenhouse study compared with greenhouse inoculation rates on the order of 1,000 infective units commonly used for migratory endoparasitic nematodes, for which reproductive factors are most commonly calculated (Davis and Webster, 2005; Grabau and Chen, 2014). Lower inoculation rates in sting nematode experiments may inflate reproductive factor values based on mathematical principles. Despite these considerations, the inoculation rates used are in line with the 50 to 200 sting nematodes per pot used in most sting nematode greenhouse trials (Khuong and Smart, 1975; Timper and Hanna, 2005; Braz *et al*., 2016) and are necessary because excess inoculation rates could result in plant death and sting nematode population crashes (Crow *et al*., 2000a). Additionally, these inoculation rates reflect carrying capacity in agricultural fields as sting nematode population densities did not surpass 200 sting nematodes/100 cm^3^ soil in a potato trial (Grabau *et al*., 2019) or cover crop trial (Crow *et al*., 2001) in Northeast Florida.

Sunn hemp was also a poor host for sting nematode based on the results of this study, similar to the limited amount of prior research (Braz *et al*., 2016). This indicates that sweetpotato or sunn hemp could be useful in a rotation for sting nematode management, although more research, particularly field trials including multiple cultivars, is needed to fully determine sting nematode population dynamics on these crops and thus sting nematode management value in a rotation scheme. In addition to field testing, assessment of sweetpotato against multiple populations or a representative population for each intended production area could be a useful future research direction. Sting nematode pathotypes exist based on prior research showing variations in the crop host status by the sting nematode population (Abugharbieh and Perry, 1970; Robbins and Barker, 1973; Robbins and Hirschmann, 1974), making this an important consideration. Despite their limitations, greenhouse studies like this are important for sting nematode host status evaluation because they make it possible to fully control important variables including initial nematode abundances, plant species present (viz., weed exclusion), and nematode species present, which is not feasible in field studies. In sum, this greenhouse study is an important step in assessing the value of these crops for sting nematode management.

Regarding pathogenicity, sting nematode did not consistently affect the growth of any crop in this study, including corn, which is known to be severely damaged by sting nematode in production settings (Rhoades, 1978; Todd, 1989). This is likely a reflection that crop tolerance to nematodes may be greater in controlled, limited-duration greenhouse testing, as opposed to stressed field settings, rather than a comprehensive assessment of sting nematode damage potential on these crops. Certainly, the fact that sweetpotato is a poor host for sting nematode makes it less likely that it is susceptible to sting nematode damage. However, this needs to be verified as crops can sustain nematode damage even if they do not support prolific reproduction of a particular nematode (Kutsuwa *et al*., 2015). Sting nematode is acutely damaging (Crow *et al*., 2000a, 2000c) and, as a migratory ectoparasite (Huang and Becker, 1999), could temporarily feed on crops that are not a preferred host, making further verification of damage potential independent of reproduction particularly important.

Lack of sting nematode damage, even to susceptible hosts, in this greenhouse study is not out of line with prior sting nematode greenhouse studies, which have varied in this respect. In greenhouse experiments on kale (*Brassica oleracea* var. *sabellica* L.), collard (*Brassica oleracea* var. *acephala* L.), and cauliflower (*Brassica oleracea* var. *botrytis* L.), sting nematode has generally reduced the growth of all crops when inoculated at 100 sting nematodes per pot (Khuong and Smart, 1975). Plant damage was less pronounced at an inoculation rate of 50 sting nematodes per pot (Khuong and Smart, 1975). In another greenhouse study, sting nematode (200 nematodes per pot) significantly decreased final root biomass for three of 11 sunn hemp cultivars but did not affect aboveground growth parameters (Braz *et al*., 2016). In a greenhouse study of the interactions of sting nematode isolates from various locations on various crops, sting nematode (100 nematodes per pot) did not consistently affect plant growth, even for suitable hosts (Abugharbieh and Perry, 1970). In a field corn greenhouse study, an initial corn crop was not affected by sting nematode at an inoculation rate of 500 or 1,000 nematodes per pot, although in a second corn crop, rotated after the first crop, either sting nematode rate decreased corn weight (Rhoades, 1985). Further research, particularly field studies, is needed to fully assess sting nematode damage potential on sweetpotato.

In summary, sunn hemp and sweetpotato were poor hosts, supporting relatively little sting nematode reproduction based on this greenhouse study. These crops could be useful rotation crops for managing sting nematode, particularly in comparison to field corn, a susceptible host. Further assessment is needed to determine sting nematode damage potential on sweetpotato as sting nematode did not affect the growth of any crop in this study, including field corn, which is known to be susceptible to damage.
